# Masked and elusive: congruency fails in video-to-photo face matching

**DOI:** 10.1186/s41235-026-00712-2

**Published:** 2026-02-18

**Authors:** Anna Sagana, Mengying Zhang, Lara Bekemeier, Evie Nelissen, Melanie Sauerland

**Affiliations:** Section Forensic Psychology, Department of Clinical Psychological Science, Faculty of Psychology and Neuroscience, Maastricht University, Universiteitssingel 40, PO Box 616, 6200 MD Maastricht, The Netherlands

**Keywords:** Masked perpetrators, Surveillance, CCTV, Forensic face matching

## Abstract

Recognizing masked perpetrators in real-world surveillance scenarios poses significant challenges due to facial occlusion and degraded image quality. This study investigated the effects of contextual congruency on matching surveillance videos to suspects’ photos. Participants (*N* = 229) completed a face-matching task involving four masked or unmasked video targets paired with either full face or masked photos. Matching accuracy was significantly higher for unmasked faces compared to masked faces, with no significant congruency effect between video and photo conditions. Participants' confidence was generally higher in congruent than incongruent conditions, particularly when viewing full-face videos. The confidence-accuracy relationship was condition-dependent, emerging as significant only when masked videos were paired with masked photographs. These findings emphasize the limitations of human performance in identifying masked individuals under degraded conditions and the constraints of potential strategies for improving face recognition in forensic and surveillance contexts.

## Introduction

Law enforcement's reliance on CCTV and other surveillance footage has increased substantially in recent decades. For example, surveillance cameras in the United States grew nearly 50% from 2015 to 2018 (U.S. Bureau of Labor Statistics, 2021), and 90% of large police departments now using video surveillance (Oliver & Kugler, 2021). This is arguably because CCTV significantly increases the likelihood of solving crimes (Ashby, 2017). The value of CCTV evidence is also demonstrated in cases such as the 2013 Boston Marathon bombing, where video analysis proved critical in identifying the perpetrators (Federal Bureau of Investigation, 2023). However, face matching from surveillance footage is highly error-prone (Bindemann et al., 2013; Burton et al., 1999; Henderson et al., 2001). The task becomes even more challenging when perpetrators obscure large parts of their faces (Zhang et al., 2025c). Combined with the poor quality of real-world recordings—low resolution, unfavorable angles, and poor lighting (Henderson et al., 2001)—this makes face matching a difficult task for investigators (Davis & Valentine, 2009). It is therefore essential to explore strategies to mitigate these challenges. The current study investigates whether contextual congruency, where matching conditions mirror the original viewing conditions (e.g., masked face–masked face), can improve face-matching accuracy for masked perpetrators in realistic, low quality, surveillance conditions for masked targets.

Contextual congruency has shown beneficial effects on face matching, recognition, and lineup identification tasks (Estudillo & Wong, 2022; Hockley et al., 1999; Manley et al., 2019, 2022; Palu et al., 2024; Sagana, 2022; Zhang et al., 2025b, 2025c). For example, when participants compare two faces and determine whether they depict the same person or different individuals, performance improved when both faces remain occluded in both pictures, compared to when one face is fully visible and the other is occluded (Estudillo & Wong, 2022; Zhang et al., 2025c). Similarly, recognition studies have found advantages when the encoded and the face to be recognized are both fully visible or both masked rather than when one face is fully visible and the other is masked (Hsiao et al., 2022; Zhang et al., 2025a, 2025b). Specifically, compared to incongruent face presentations, congruent face presentations have been shown to produce medium to large effect sizes (*η*_*p*_^*2*^ between.28 and.63), with increases in recognition sensitivity ranging from 21 to 35% (Zhang et al., 2025c). When congruency effects emerge, they persist even under heavy occlusion, where only minimal features, such as the eye or mouth region, are visible (Manley et al., 2019, 2022; Sagana, 2022; Zhang et al., 2025a). These findings suggest that the alignment between the two viewing conditions reduces the cognitive disruption caused by mismatched contexts and increases sensitivity to diagnostic facial features.

However, the effect of contextual congruency is more nuanced than these findings might suggest. Discriminability does not always differ between congruent and incongruent masked conditions (Garcia-Marques et al., 2022; Guerra et al., 2022), congruency benefits for target-present identification may not extend to target-absent rejections (Manley et al., 2022; 2024) and congruency effects may be largely absent when encoding occurs with masked faces (Palu et al. 2024). Additionally, congruency effects are stronger in perceptual face matching (Zhang et al., 2025c) than memory-based recognition tasks (Zhang et al., 2025a, 2025b). In conclusion, congruency effects may be effective only under specific task parameters.

Several mechanisms may explain these variable congruency effects. First, disguise and masking effects depend critically on the degree to which diagnostic facial features remain visible during encoding and retrieval (Mansour et al., 2020). From this perspective, contextual congruency may facilitate performance only when sufficient or specific features survive the occlusion. This suggests a hierarchical relationship where diagnostic facial feature availability serves as a prerequisite for congruency benefits to emerge. Second, the cognitive processes underlying different tasks may moderate congruency effects. Face-matching tasks, where both images are simultaneously present, rely more heavily on image-level similarity judgments (Menon et al., 2015). As such, they may be more sensitive to contextual alignment compared to memory-based tasks. Face-recognition tasks that rely on memory-based identity representations, on the other hand, may be more vulnerable to disruption from both contextual mismatch and insufficient feature encoding. Third, congruency benefits may depend on available processing strategies. When both faces are similarly occluded, observers can utilize feature-based processing, analyzing individual facial features separately rather than processing the face as an integrated whole (Leder & Carbon, 2005; Megreya & Bindemann, 2018; Towler et al., 2021). Incongruent presentations instead force observers to reconcile different strategies: holistic processing for the full face versus feature-based processing for the masked face. This shift from holistic to feature-based processing may itself be moderated by viewing conditions. When image quality is high, observers can effectively deploy feature-based strategies even with masked faces, but when quality is degraded, the remaining features may provide insufficient information for either processing strategy to succeed (see Zhang et al., 2025b).

These theoretical considerations suggest that congruency effects should be most constrained under conditions that combine limited feature availability (masking) with degraded perceptual input (poor image quality); precisely the conditions typical of real-world surveillance footage. Yet, much of the existing research has explored contextual congruency using unrealistic, laboratory-based conditions (Estudillo & Wong, 2022; Freud et al., 2020; Hsiao et al., 2022). Participants are presented with high quality, frontal images of faces at optimal viewing angles. However, in real-world surveillance images are degraded by pixilation, poor contrast, and suboptimal viewpoints. While matching familiar faces even with low-quality footage can achieve reasonable accuracy (> 73%; Burton et al., 1999), matching unfamiliar individuals is far more error-prone (Burton et al., 1999, 2010; Henderson et al., 2001). Recognition performance under suboptimal viewing conditions can be particularly poor for unfamiliar faces, with accuracies as low as 29% for CCTV images compared to 64% for higher quality stills (Henderson et al., 2001). Recent work further underscores the challenges of recognizing masked targets under suboptimal viewing conditions resembling real-world scenarios (Zhang et al., 2025b). Across two experiments, recognition accuracy for masked faces did not surpass 60%, even with contextual congruency between encoding and recognition phases. While congruent face presentations show some improvement compared to incongruent ones, overall accuracy remained alarmingly low, ranging from 47 to 60%. These findings add to earlier evidence that pixilation and low image quality impair recognition performance (Burton et al., 2010; Noyes & Jenkins, 2017).

Beyond the aforementioned challenges, the interaction between masking and image quality introduces additional complexity. Masks primarily reduce quantity by occluding features, while CCTV degradation reduces quality through pixelation and low frame rates. Interestingly, Zhang et al. (2025b) found that image quality might exert a more pronounced effect on full face recognition than on masked face recognition. This suggests that when masks limit the quantity of available information, further reductions in image quality may have less additional impact. That is, a floor effect where performance is already constrained by occlusion. However, these dimensions can also interact: even when masks reveal few features (low quantity), those features can vary greatly in quality, further complicating face recognition under real-world surveillance conditions.

Another key limitation of many face-matching studies is their reliance on static images, whereas real-world crimes often involve dynamic visual input from surveillance cameras and smartphones. Unlike static images, videos capture temporal information, such as motion, changes in facial expression, head orientation, and lighting. This dynamic display can be both beneficial and challenging for face matching. On the one hand, motion provides visual cues that can help observers perceive a face more holistically, with facial movements like blinking, speaking, or subtle shifts in expression highlighting diagnostic facial features (Lander et al., 1999; O'Toole et al., 2002). Videos may therefore offer a more accurate representation of natural facial variability (Bennetts et al., 2015; Burton et al., 2005; Jenkins et al., 2011; Young & Burton, 2017), allowing the viewer to sample the face more effectively. Indeed, some studies show improved performance in recognizing faces with dynamic stimuli (Butcher et al., 2011; Lander & Bruce, 2003; Schiff et al., 1986). On the other hand, videos can introduce motion blur and temporal inconsistencies (Burton et al., 2015; Davis & Valentine, 2009; Strathie & McNeill, 2016), especially under poor recording conditions (e.g., low frame rates or sudden movements). These distortions can make it difficult to extract stable visual information, often reducing accuracy (Burton et al., 2015; Christie & Bruce, 1998; Lander et al., 2016) or providing no improvement (Darling et al., 2008; Knight & Johnston, 1997; Mileva & Burton, 2019). Given the uncertainty surrounding the role of motion on recognition accuracy, it remains unclear whether the benefits of contextual congruency seen with still images persist under dynamic, real-world conditions.

While much of the research has focused on accuracy, there is also growing interest in how contextual congruency affects confidence and its relationship with accuracy. Studies with masked targets have shown that congruent face pairs elicit more confident responses than incongruent face pairs (Stephens et al., 2017; Zhang et al., 2025c). When viewing conditions match, as in congruent presentations, observers may experience greater processing fluency, resulting in more confident judgments (Mansour et al., 2020). From this perspective, contextual congruency might improve the confidence-accuracy relationship by ensuring matched processing strategies and reducing the cognitive disruption caused by mismatched viewing conditions. However, the relationship between confidence and accuracy under masked conditions is less clear. Some studies do not demonstrate a clear confidence-accuracy relationship under masked conditions (Manley et al., 2019; Sagana, 2022), and others suggest that confidence-accuracy correlations are limited to unmasked faces (Hsiao et al., 2022). When features are severely limited or task difficulty is high the subjective feeling of fluency may decouple from actual recognition accuracy, leading to miscalibration. Taken together, these findings suggest that confidence may not be a reliable indicator of accuracy for masked face recognition. Following up on these findings, the secondary aim of the current study is to explore the confidence-accuracy relationship for masked faces under realistic, dynamic, occluded conditions.

### The current study

To address the limitations of prior research, the present study investigated the role of contextual congruency in video-to-photo face-matching tasks involving masked perpetrators. Specifically, participants viewed low-quality mock crime videos of masked or unmasked perpetrators and were tasked with matching these perpetrators to suspect photos that displayed either a full face or a masked face.

The current study draws on feature-based processing as its theoretical framework (Leder & Carbon, 2005; Megreya & Bindemann, 2018; Towler et al., 2021). We reasoned that contextual congruency should improve performance by reducing reliance on incomplete holistic representations and instead directing attention to the most informative, consistently visible features. Beyond performance, the shift from holistic to feature-based processing may also influence response bias. Prior research suggests that disguises systematically affect decision criteria, with masked faces often eliciting more conservative responding (Carragher & Hancock, 2020; Estudillo et al., 2021). Incongruent viewing conditions, where participants may alternate between holistic and feature-based processing, may increase processing difficulty and lead to more conservative decision criteria. Conversely, congruent conditions that facilitate feature-based processing may reduce this processing difficulty and associated decision uncertainty. As such, we hypothesized that contextual congruency would improve matching performance relative to incongruent conditions for both masked and unmasked perpetrators (H1). Regarding confidence, we expected higher confidence in congruent than incongruent matching conditions (H2), higher confidence in accurate than inaccurate matches (H3), and a stronger confidence-accuracy relationship in congruent than incongruent matching conditions (H4).

## Methods

The experiment received ethical approval from the standing Ethics Review of our faculty (ERCPN-OZL_245_155_11_2021) and it was pre-registered on the Open Science Framework (https://osf.io/y64vr). We note one deviation from the pre-registration. We did not conduct exploratory gender analyses, as the relevant interactions would be severely underpowered (approximately 16 participants per cell). The datasets generated and analyzed during the current study are available in the Dataverse repository (10.34894/8OU1VT).

### Participants

Our target sample size was 268 participants, with the aim of achieving a power of.95 to detect a medium effect size (*OR* = 2.4) at the standard.05 alpha error probability. We recruited 286 participants via *Prolific* and personal networks to account for dropouts. Participants who provided incomplete data (34 participants), reported familiarity with any of the video targets or photos (12 participants), or failed the attention check (3 participants) were excluded from the final analysis. Thus, the final sample consisted of *N* = 229 Caucasian participants (123 female, 105 male, 1 undisclosed) with a mean age of 34.4 years (*SD* = 13.5). The actual power of the study, given the final sample size, is approximately 93% and the study remains well powered to detect a medium effect size (*OR* = 2.4) at α = 0.05. Participants were recruited predominantly via Prolific (*n* = 183) and compensated at the platform's standard rate (2.50€), with a smaller subset (*n* = 46) recruited through personal networks without monetary compensation.

### Design

The study employed a mixed 2 (video: full face vs. masked face; between-subjects) × 2 (photo: full face vs. masked face; between-subjects) × 2 (trial type: match vs. mismatch; within-subjects) design. To explore the effects of masks on matching performance, we compared the proportion of correct responses (i.e., accuracy), sensitivity (i.e., *d*-prime) and response bias (i.e., criterion *c*) across the full-view and mask conditions. Sensitivity and response bias were calculated using signal detection theory, applying the 1/(2*N*) correction. Higher *d*-prime values indicate better ability to discriminate between matching and non-matching pairs, with 0 representing chance-level performance. For criterion *c*, negative values indicate a tendency to respond "same person" (i.e., liberal response bias), positive values indicate a tendency to respond "different person" (i.e., conservative response bias), and 0 represents a neutral criterion with no systematic bias. Confidence was measured using a sliding scale from 0 (= *not confident at all)* to 100 (= *absolutely positive*).

### Materials

#### Mock crime CCTV videos and face pairings

**Fig. 1:**
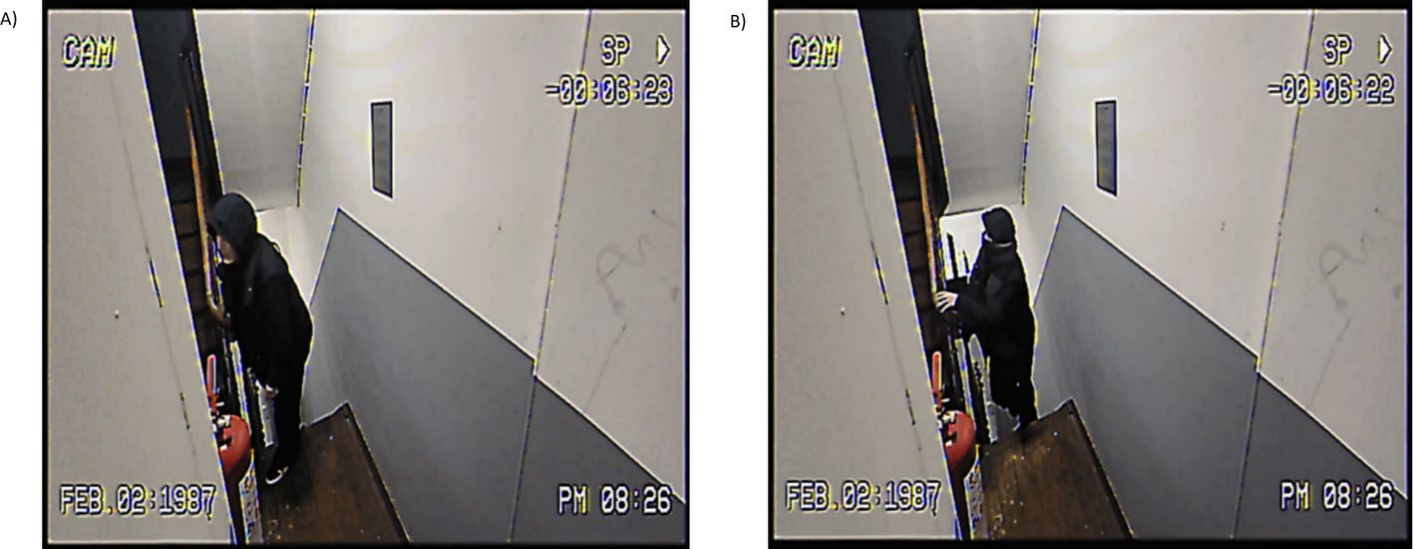
Example of the video footage used in the experiment. Panel A presents a video featuring an unmasked target, while Panel B presents its masked equivalent. *Note:* In the examples, the target's face is deliberately kept hidden. However, the videos show clear frontal and lateral views of all targets

We recorded several versions of four mock crimes (i.e., home invasion, bike theft, purse theft, and ATM theft), using an *iPhone 14 Pro*. We recorded each mock crime featuring both a male and a female perpetrator, either with each perpetrator wearing a scarf and hat that obscured all facial features except the eyes or with the full face visible. This approach generated four versions of each crime (masked male, unmasked male, masked female, unmasked female), resulting in 16 distinct video recordings. Figure 1 shows an example of video footage. To mimic the perspective typical of CCTV cameras, the videos were filmed from an upper corner using a wide-angle zoom. To mimic the quality typical of CCTV footage, video quality was reduced using the software *CapCut.* We applied the "Low Quality" filter, reduced the frame rate to four frames per second (4fps), and layover a "Retro Cam" filter to add a realistic camera frame. While 4fps is below current CCTV standards, it represents degraded or storage-constrained surveillance conditions that have been documented in practice, particularly in older or budget systems. The duration of the videos ranged from 20 to 71 s and included no sound. Targets remain visible for the entire duration of the videos.

To create matching pairs, we coupled each video with a (same or different) static photo. In creating same person (i.e., matched) pairs, we took targets’ photos under standardized conditions (i.e., neutral expression, good lighting, white background, hair worn loose tucked behind the ear, and no jewelry). In creating different people (i.e., mismatched) pairs, filler photos were sourced from our standardized Face Database. Three of the authors selected the initial filler options (2–3 alternatives per face). Initial selections ensured that fillers shared basic facial characteristics with the perpetrators, including hair color and face shape. No additional contextual factors were considered. Subsequently, a pilot study (*N* = 28) was conducted to refine the filler-target pairings. Participants in the pilot study evaluated the similarity, dissimilarity, and distinguishability of all potential pairs, on a scale from 0 to 100, and selected the filler that most closely resembled the video target. We opted for pairings that showed moderate levels of similarity to avoid making the task overly difficult. The mean similarity ratings of the selected pairings are *M* = 51.31 (range 44.22 to 70.47) and the mean dissimilarity is* M* = 45.51 (range 27.53 to 57.88). Once the target and filler photos were selected, we created two versions for each photo: one displaying the full face and one displaying only the eye region by covering the forehead, mouth, and chin with black blocks (for similar approach, see Zhang et al., 2025a, 2025c).

### Procedure

We tested participants online. After entering their demographic details, participants were assigned randomly to one of four experimental conditions. In each condition, each participant viewed one video per crime type (home invasion, bike theft, purse theft, ATM theft), in random order, resulting in four different mock crime videos. The perpetrator's face was either covered or remained unmasked. Beneath each video, participants could see a suspect’s photo. Depending on the assigned condition, the photo displayed either the full face or the masked face. Within each experimental condition, participants completed two match trials and two mismatch trials, with the match/mismatch assignment randomized across the four crimes. Beneath each video, participants viewed a single suspect photo and decided whether the suspect’s photo matched the perpetrator in the video. They responded with either “*Yes, same person”* or “*No, different person”*. Participants could re-watch or pause the videos as often as they wished, without any limitations, just as they would in real life. After each decision, participants rated their confidence in their decision before they could see the next video and photo pairing. Finally, participants were debriefed and thanked for their participation. The entire procedure took approximately 10–15 min to complete.

## Results

Table 1 presents descriptive statistics for matching performance and post-decision confidence. Matching accuracy was low in all conditions but significantly above chance level (50%), all *ts* (229) ≥ 2.17, *ps* ≤.034, *ds* ≥ 0.60. Accuracy was highest in the congruent full face video – full face photo condition. Contrary to expectations, accuracy was lowest in the congruent masked video – masked face photo condition.

**Table 1:** Descriptive statistics for matching accuracy, sensitivity (d’), response bias (c) and confidence

Video condition	Photo condition	Accuracy	Sensitivity* (d’)*	Response bias *(c)*	Confidence
		*M (SE*)	*M (SE*)	*M (SE*)	*M (SE*)
Full Face†	Full Face	.74 (.03)	1.09 (1.10)	− 0.24 (0.51)	66.81 (1.41)
Full Face‡	Masked	.65 (.03)^a*^	0.69 (1.16)	0.11 (0.49)^a***^	55.80 (1.46)^a***d***^
Masked‡	Full Face	.59 (.03)^a***^	0.40 (1.34)^a**^	− 0.03 (0.47)^a***d*^	52.76 (1.65)^a***d**^
Masked†	Masked	.57 (.03)^a***^	0.33 (1.16)^a***^	0.20 (0.57)	58.97 (1.49)^a**^

Conditions are ranked from higher to lowest matching accuracy. The mean sensitivity and response bias values are calculated using the *1/(2N)* correction. † indicates contextually congruent testing conditions, ‡ indicates contextually incongruent testing conditions. The superscript letters indicate significant differences from ^*a*^ = full-full, ^*b*^ = full-masked, ^*c*^ = masked-full, ^*d*^ = masked-masked, at *p <.05, **p <.01, ***p <.001

### Video to face matching performance

To investigate the effects of video condition (full face vs. masked face), photo condition (full face vs. masked face), and their interaction on matching accuracy, we conducted a Generalized Linear Mixed Model (GLMM) using JASP with a logit link function and maximum likelihood (ML) estimation. Participants were included as a random effect. Contrary to predictions (H1), the interaction effect between video and photo conditions was not significant, *Wald χ*^*2*^*(1)* = 1.19*, p* =.551, *OR* = 1.40, *95% CI* [0.81, 2.42]. After removing the non-significant interaction term, the analysis revealed a significant main effect of video condition, *Wald χ*^*2*^(1) = 13.16, *p* <.001, *OR* = 1.65, *95% CI* [1.25, 2.16]. Accuracy was higher for videos depicting unmasked targets (*M* =.69, *SE* =.02) compared to masked targets (*M* =.58, *SE* =.02). The main effect of photo condition was not significant, *Wald χ*^*2*^(1) = 2.92, *p* =.232, *OR* = 1.26, *95% CI* [0.97, 1.66]. Therefore, the effect of the video condition on accuracy was independent of the photo condition.

As recognition accuracy provides a rather crude measure of performance, we also examined the effect of video condition and photo condition on sensitivity and response bias. We conducted two separate ANOVAs with video condition, photo condition, and their interaction as factors. Similar to accuracy, the interaction effect did not reach significance, *F*(229,1) = 1.01, *p* =.316, *η*_*p*_^*2*^ <.01 and neither did the main effect of photo, *F*(229,1) = 2.27, *p* =.134, *η*_*p*_^*2*^ =.01. However, there was a significant main effect of video on sensitivity, *F*(229,1) = 11.18, *p* <.001, *η*_*p*_^*2*^ =.05. Participants were better able to discriminate between matched and mismatched pairs when the video displayed an unmasked (*d’* = 0.89, *SD* = 1.14) than a masked perpetrator (*d’* = 0.36, *SD* = 1.24).

Regarding response bias, the interaction term did not attain statistical significance, *F*(229,1) = 0.76, *p* =.383, *η*_*p*_^*2*^ <.01. The main effect of the video, *F*(229,1) = 5.01, *p* =.026, *η*_*p*_^*2*^ =.02, and the photo condition, *F*(229,1) = 18.73, *p* <.001, *η*_*p*_^*2*^ =.08, were both significant. Participants used a more liberal criterion when the videos depicted unmasked (*c* = − 0.06, *SD* = 0.53) rather than masked (*c* = 0.09, *SD* = 0.53) targets, but more so when the photo to be matched depicted a full face (*c* = − 0.14, *SD* = 0.50) rather than a masked face (*c* = 0.15, *SD* = 0.53).

Taken together, the results do not support the hypothesis of a contextual congruency effect in dynamic video-photo matching (H1). The only consistent factor influencing performance was the presentation of the face in the video, with higher accuracy and sensitivity for unmasked videos compared to masked ones. While the photo condition alone did not significantly influence accuracy or sensitivity, its significant effect on response bias highlights its role in shaping participants' decision-making strategies.

### Post-decision confidence

A linear mixed model (LMM) was fit using SPSS with restricted maximum likelihood (REML) estimation to predict confidence from video condition, photo condition, accuracy, and their interactions, with random intercepts for participants to account for repeated measurements. Factors that made no significant contribution to the model were removed sequentially from highest to lowest order (i.e., interactions before main effects). Table 2 provides an overview of the inferential statistics of the final model. Given the presence of a significant three-way interaction between video condition, photo condition and accuracy (see Table 2), we report parameter estimates separately for full face and masked face video conditions. In the full face video condition, the interaction between accuracy and photo condition did not reach significance, *B* = 5.45, *SE* = 4.02, *F*(1, 415.32) = 1.83, *p* =.177. After removing the non-significant interaction term, both the main effect of photo condition, *B* = − 10.66, *SE* = 2.88, *F*(1, 113.65) = 13.66,* p* <.001, and accuracy, *B* = − 4.08, *SE* = 2.01, *F*(1, 418.12) = 4.11, *p* =.043**,** became significant predictors of confidence. When seeing a congruent full face photo, participants’ confidence was considerably higher (*M* = 66.81, *SE* = 1.41) than when seeing an incongruent masked face photo (*M* = 55.08, *SE* = 1.46). Additionally, participants showed a modest increase in confidence when making accurate (*M* = 62.68, *SE* = 1.27) than inaccurate decisions (*M* = 58.03, *SE* = 1.82). Overall, these findings suggest that video to photo congruency plays a critical role in shaping confidence in full-face conditions, but the relationship between accuracy and confidence, while present, is relatively weak.

**Table 2:** Fixed effects and parameter estimates for final LMM predicting confidence from video, photo, accuracy and interaction terms

	Estimates of fixed effects							Fixed effects	
	*b* coefficien*t*	*SE*	*95% CI* (Lower)	*95% CI* (Upper)	*df*	*t*	*p*	*F*	*p*
*(Intercept)*	68.69	2.31	64.15	73.24	279.78	29.74	<.001	2759.88	<.001
Video	− 16.16	3.42	− 22.89	− 9.43	308.77	− 4.72	<.001	4.68	.031
Photo	− 12.34	3.30	− 18.83	− 5.84	293.97	− 3.74	<.001	0.84	.360
Accuracy	− 7.14	3.07	− 13.17	− 1.11	826.59	− 2.33	.020	9.37	.002
Video × Photo	22.78	4.79	13.35	32.21	316.76	4.75	<.001	11.63	.001
Video × Photo × Accuracy	2.76	–	–	–	828.85	–	–	2.76	.041

Estimates are based on a linear mixed model predicting confidence, with random intercepts for participants

*SE* standard error, *df* degrees of freedom, *CI* confidence interval

In the masked video condition, there was a significant interaction effect between accuracy and photo condition, *B* = − 9.87, *SE* = 4.03*, F*(1, 414.9) = 5.99,* p* =.015. Follow-up models split by photo condition revealed that, when viewing a congruent masked face photo, accuracy was a strong predictor of confidence, *B* = − 9.38, *SE* = 2.56, *F*(1, 208.81) = 13.44, *p* <.001. Participants were more confident in accurate (*M* = 62.67, *SE* = 1.90) than inaccurate decisions (*M* = 54.07, *SE* = 2.31). However, when viewing incongruent full face photos, participants were equally confident in their accurate (*M* = 51.61, *SE* = 2.14) and inaccurate decisions (*M* = 54.43, *SE* = 2.62), *B* = 0.67, *SE* = 3.15, *F*(1, 203.79) = 0.04, *p* =.832. Hence, participants' confidence was significantly associated with accuracy only under congruent conditions, where both the video and photo depicted masked faces.

Taken together, these findings provide partial support for our hypotheses. Consistent with H2, confidence was generally higher in congruent than incongruent conditions, particularly when participants viewed a full face video. However, this effect was not uniform across conditions, as confidence in the masked video condition was similar regardless of photo congruency. As expected, confidence was significantly higher for accurate than inaccurate matches (H3), particularly in the full face video condition. However, the anticipated stronger confidence-accuracy relationship in congruent conditions (H4) was present only in the masked video condition, where participants’ confidence was significantly associated with accuracy when both the video and photo depicted masked faces. This suggests that the relationship between confidence and accuracy is not uniform but depends critically on visibility conditions and their congruency.

### Confidence-accuracy relationship

**Fig. 2:**
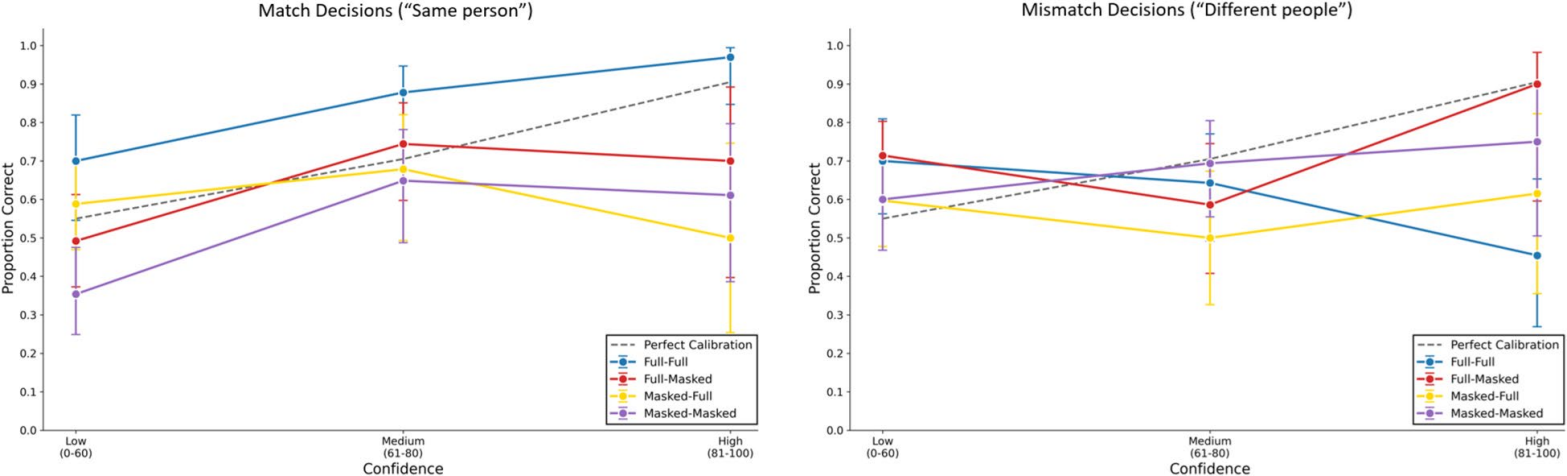
Confidence Accuracy Curves (CAC) for match (“same person”, upper panel) and mismatch (“different people”, lower panel) decisions across the four experimental conditions. *Note*. Error bars show 95% confidence intervals for the proportions

We created separate confidence accuracy curves for match (“same person”) and mismatch (“different people”) decisions. For the CAC analysis, we plotted the proportion of correct matching decisions across thee bins of confidence (low: up to 60%, medium: 61 − 80%, high: 81 − 100%). Figure 2 shows that participants’ confidence ratings were not well-calibrated either for match (“same”) or for mismatch (“different”) decisions. Only exception are participants who made match decision in the full-full condition. Notably, the CAC was lowest when a masked face was paired with a full-face (masked-full condition). These results are consistent with our findings on matching performance in failing to reveal a consistent congruency effect and suggest that witness confidence should be treated with particular caution in cases involving masked perpetrators.

**Fig. 3:**
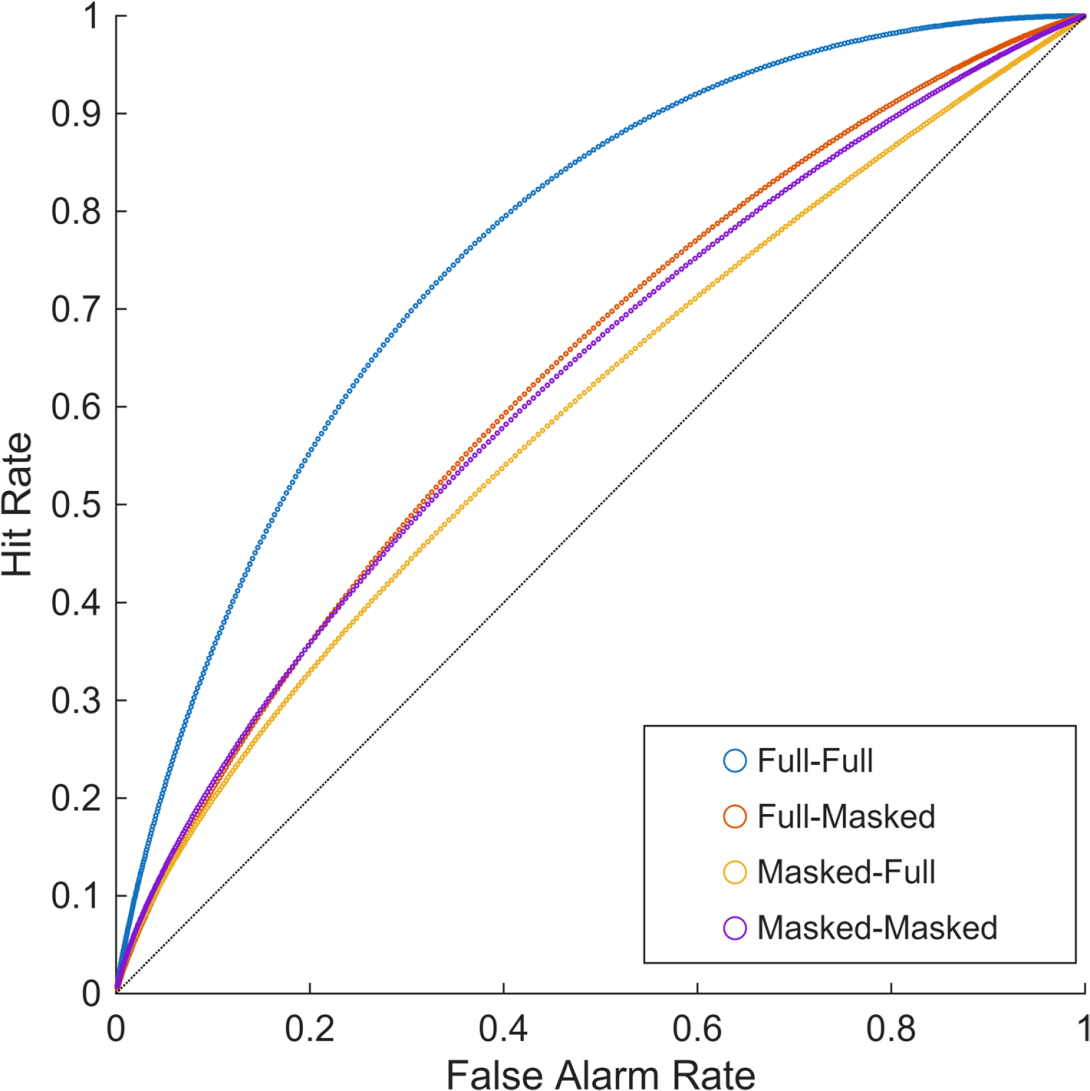
Receiver Operating Characteristic (ROC) curves for the four experimental conditions. *Note.* AUC values were: Full-Full =.764, Full-Masked =.645, Masked-Full =.601, Masked-Masked =.629

The ROC analysis revealed substantial differences in discriminability across conditions (Fig. 3). Seeing the full face of the target both in the video and in the photo (full-full condition) yielded the best discrimination ability (AUC =.76), significantly outperforming all other conditions (all *p*s ≤.046). The three conditions involving masking showed significantly poorer discriminability, with no significant differences among them, all AUCs ≤.64, all *ps* ≥.499. These findings indicate that the presence of masking in either the video or photo substantially impairs discrimination, but the specific location of masking (video versus photo) or contextual congruency did not significantly differentiate performance once masking was introduced.

## Discussion

This study investigated the effects of contextual congruency on video-to-photo face matching for masked perpetrators under suboptimal, real-world viewing conditions. Contrary to expectations, the beneficial effects of congruency on matching performance and confidence were limited, with the video condition exerting a stronger influence. Videos of unmasked targets were associated with higher matching performance and confidence, while congruency between video and photo conditions did not significantly enhance these outcomes. This suggests that congruency between video and photo may not be as influential as previously established in dynamic displays.

Contrary to expectations (H1), accuracy for masked video conditions remained alarmingly low (ranging from 57 to 59%) even in congruent scenarios. These modest congruency effects observed align with prior research indicating that congruency effects weaken under degraded conditions (Zhang et al., 2025b). The only factor that exerted a significant influence on matching accuracy was the masking of targets in the video. Face-matching performance was higher for unmasked compared to masked video targets and participants were better able to discriminate the faces when the videos depicted unmasked faces. These results emphasize that recognition primarily depends on the quantity and quality of available visual information rather than contextual congruency.

Furthermore, response bias analyses showed that participants used stricter decision criteria when viewing videos of masked faces, regardless of whether the matching face was masked or unmasked. This aligns with previous studies showing that people are more likely to declare mismatches when a face is masked (Estudillo et al., 2021; Zhang et al., 2025c). Hence, participants seem to adopt a more conservative strategy in these conditions. Looking more closely at the masked face video condition, participants showed a near-neutral bias if the comparison face was unmasked but adopted a more conservative criterion if the comparison face was also masked (see Table 1). This contrasts with previous reports of consistently conservative bias when matching masked faces to full faces (Carragher & Hancock, 2020; Estudillo et al., 2021). The difference may be due to dynamic video cues and the detailed features in full face images, which provide more information for making "match" decisions (Dobs et al., 2018). The limited impact of congruency suggests that under suboptimal conditions, such as masked faces and degraded visual quality, congruency effects may be overshadowed by the challenges posed by heavy occlusion.

Taken together, our findings suggest that the beneficial effects of contextual congruency cannot compensate the undermining effects of limited feature availability with low image quality. Hence, congruency effects might operate as secondary processes that can optimize performance only when sufficient facial information is available for face recognition. This hierarchical relationship between information availability and contextual factors aligns with perceptual models proposing that face processing follows a specific sequence: first extracting available facial features, then integrating them with contextual information (Bruce & Young, 1986; Rhodes, 1988; Schyns et al., 2002). These findings also align with Mansour et al.'s (2020) proposal that disguise effects depend on the degree to which critical features remain accessible during encoding and retrieval. Notably, face-matching tasks may facilitate congruency effects more than memory-based recognition tasks, because no representation of identity needs to be created when both faces are visible simultaneously (Menon et al., 2015). However, the present findings, along with work using a memory-based recognition paradigm (Zhang et al., 2025b) under similar degraded conditions, suggest that insufficient visual information constrains congruency benefits regardless of task structure. In our study, the combination of heavy occlusion and degraded video quality likely reduced feature accessibility below the threshold necessary for congruency to enhance performance.

At a secondary level, the lack of congruency effects in dynamic displays could be explained by the notion that face perception depends on two separate but interactive pathways (Duchaine & Yovel, 2015). One pathway extracts structure and surface properties of a face and matches these with stored representations, while the other represents aspects of faces that change rapidly, such as expression, gaze, and mouth movements. This parallel, interactive processing requires both pathways to have sufficient input to operate effectively. In our study, we combined static pictures with videos of suboptimal quality, which may explain why we could not see benefits—the motion pathway may have lacked sufficient input to enhance face processing.

Regarding post-decision confidence and the confidence-accuracy relationship, the findings provide mixed support for our hypotheses. Contextual congruency between video and photo conditions had a moderate enhancing effect on confidence (H2), especially in the full face video condition. This suggests that congruency alone can be a determinant of metacognitive judgements. Confidence was also generally higher for accurate than inaccurate matches (H3), but this effect was not uniform across conditions. The anticipated stronger confidence-accuracy relationship in congruent conditions (H4) was also only partially supported. Confidence-accuracy relationships were reliable in full-face conditions (regardless of congruency), but they weakened in masked scenarios, supporting earlier observations (Hsiao et al., 2022; Palu et al., 2024). Notably, for masked faces, confidence was associated with accuracy only under congruent testing conditions, indicating that congruency becomes critical for confidence judgments when facial information is limited. These results align with the calibration framework of Wixted and Wells (2017), which posits that confidence-accuracy relationships deteriorate as discriminability decreases. For masked faces with inherently lower discriminability, confidence becomes a poor predictor of performance, a concerning issue in applied settings. Overall, our findings corroborate previous research showing confidence is not consistently reliable under suboptimal conditions (Manley et al., 2019; Sagana, 2022; Zhang et al., 2025c) and highlight the complex relationship between confidence and accuracy for masked targets that warrants further investigation.

### Contextual congruency and real-world implications

The modest contextual congruency effects observed in this study raise questions about the practical value of congruency in surveillance contexts. While contextual congruency generates robust effects in idealized settings (Estudillo & Wong, 2022; Zhang et al., 2025c), its effectiveness is constrained by degraded visual input (see Zhang et al., 2025a, for similar findings). These findings carry significant implications for forensic and legal contexts, particularly when relying on surveillance footage for identifying individuals. The significant drop in accuracy for masked perpetrators highlights the inherent challenges in human face recognition under such conditions. Masking reduces the availability of critical facial features, complicating the task and increasing the likelihood of errors. This underscores the need for forensic protocols that carefully consider the limitations of human performance, especially when masked targets are involved.

Furthermore, the inconsistent confidence-accuracy relationship observed here and elsewhere (Sagana, 2022; Zhang et al., 2025a, 2025b, 2025c) emphasizes the limitations of contextual congruency in facilitating face matching under challenging viewing conditions. This is especially relevant in the context of masked perpetrators, where the uncertainty introduced by limited facial cues weakens the predictive value of confidence ratings. Our results caution against overreliance on confidence in such cases. Moreover, our findings suggest that confidence-based decision thresholds should be adjusted differently for masked versus unmasked faces in security applications, with higher thresholds potentially required for decisions involving masked faces to achieve comparable reliability levels.

### Limitations and future directions

While this study simulated real-world surveillance conditions, it may not fully capture the complexity of such environments, including dynamic distractions or time pressure. These constraints could shift decision criteria in negative directions and degrade overall performance. For example, pressure to identify suspects quickly could lead to more liberal criteria and increased false positive errors. Conversely, security personnel working under stress or fatigue might adopt more conservative thresholds to avoid false alarms, potentially missing critical identifications. Additionally, we did not track how frequently participants replayed the videos, which could have provided insight into viewing strategies across conditions. Future research could examine whether differential viewing patterns emerge for masked versus unmasked stimuli and how these relate to matching performance.

Furthermore, although accuracy was above chance in all conditions, performance remained suboptimal. The degree of degradation is likely a decisive factor in face-matching performance, with less degradation potentially yielding higher accuracy and a greater likelihood of observing congruency effects. The present study employed a single level of video degradation, but as the quality of surveillance system pictures continuously improves, future research could manipulate degradation levels systematically to explore this relationship. The use of untrained participants may have also contributed to suboptimal performance. However, while some studies have demonstrated better performance by specialist personnel in security services (Phillips et al., 2018; Robertson et al., 2016), untrained individuals and specialists, such as police and passport officers, often perform similarly on unfamiliar face tasks (White et al., 2015). Notably, even specialist performance remains imperfect, suggesting that the improvements identified here could have practical operational benefits. Future research should examine how task complexity and professional training influence performance in masked face matching and explore advanced technological solutions to support human decision-making in this context.

## Conclusion

This study highlights the limited role of contextual congruency in improving video-to-photo face-matching accuracy for masked targets. While full face video conditions significantly enhanced performance and confidence, congruency between video and photo stimuli did not yield substantial benefits. These findings emphasize that the availability and quality of visual information, rather than congruency, play a more critical role in real-world scenarios. The challenges posed by facial masking underline the need for cautious reliance on human judgments in forensic and surveillance contexts. By highlighting the challenges of masked face recognition, this study provides valuable insights for advancing forensic practices in surveillance contexts.

## Data Availability

The datasets generated and analyzed in the current study are available in the Dataverse repository [10.34894/8OU1VT].
